# Development and Physicochemical Characterization of Rice Bran Oil Oleogels Structured with Beeswax, Carnauba Wax, and Their Blends

**DOI:** 10.3390/gels12060532

**Published:** 2026-06-13

**Authors:** Ali Yassoralipour, Lorraine Ruo-Yuen Ng, Guanghui Li, Mas Munira Rambli, Sook Wah Chan, Lye Yee Chew, Nang Htet Hnin Htwe, Eng-Tong Phuah

**Affiliations:** 1 Department of Agricultural and Food Science, Faculty of Science, Universiti Tunku Abdul Rahman, Kampar 31900, Perak, Malaysia; aliyas@utar.edu.my (A.Y.);; 2 College of Light Industry and Food Sciences, Zhongkai University of Agriculture and Engineering, Guangzhou 510225, China; liguanghui@zhku.edu.cn; 3 Department of Food Science and Technology, School of Applied Sciences and Mathematics, Universiti Teknologi Brunei, Bandar Seri Begawan BE1410, Brunei; masmunira.rambli@utb.edu.bn; 4 School of Biosciences, Faculty of Health and Medical Sciences, Taylor’s University, Subang Jaya 47500, Selangor, Malaysia; sookwah.chan@taylors.edu.my (S.W.C.); lyeyee.chew@taylors.edu.my (L.Y.C.);; 5 Centre for Active Living, Taylor’s University, Subang Jaya 47500, Selangor, Malaysia

**Keywords:** rice bran oil, oleogels, beeswax, carnauba wax, hybrid wax systems, physicochemical properties, rheology

## Abstract

Oleogels have emerged as promising alternatives to conventional solid fats by structuring liquid oils without increasing trans or saturated fat levels. This study therefore aimed to develop rice bran oil (RBO)-based oleogels using beeswax (BW), carnauba wax (CW), and their combinations, and to compare their physicochemical properties with commercial margarine. Thirteen formulations with varying wax concentrations were prepared and analyzed using differential scanning calorimetry, microscopy, rheology, texture profile analysis, oil binding capacity, slip melting point, peroxide value, color analysis, and fatty acid profiling. Our results demonstrated that the thermal behavior of the oleogels is dependent on the type and concentration of the wax, with CW oleogels exhibiting higher crystallization and melting temperatures than BW, while hybrid systems displayed intermediate and synergistic properties. Distinct crystal morphologies were observed, with BW forming needle-like and CW forming spherulitic structures, while the hybrids created interconnected networks. All samples exhibited shear-thinning and gel-like behavior, with greater viscosity and gel strength observed at increasing wax concentrations. The hybrid oleogels achieved hardness comparable to higher CW levels and approached margarine texture, while maintaining high oil binding capacity (>94%). The RBO oleogels contained higher unsaturated fatty acids but showed lower oxidative stability than margarine. Overall, BW–CW hybrid oleogels demonstrated strong potential as healthier, solid fat alternatives with improved structural and thermal characteristics.

## 1. Introduction

Oils and fats derived from animal and plant sources are essential ingredients in many food products, including fried foods, baked goods, salad dressings, and spreads. Beyond serving as energy sources, they contribute significantly to mouthfeel, texture, aroma, and overall sensory appeal [1]. However, industrial processes such as partial hydrogenation, which are used to convert liquid oils into semi-solid fats, unintentionally produce trans fatty acids that have been linked to adverse health effects [2]. This has led to increasing efforts to develop healthier fat alternatives that can replicate the functional properties of solid fats without elevating trans or saturated fat levels.

One promising approach is the development of edible oleogels. Oleogels can be formed by physically organizing liquid oils, which are rich in unsaturated fatty acids, into semi-solid materials without chemically altering their composition [3]. This process, also known as oleogelation, involves incorporating an oleogelator into liquid oil. The role of an oleogelator is to form a three-dimensional network that entraps and immobilizes oil molecules, thereby transforming the liquid oil into a stable semi-solid structure [4]. The type and concentration of the oleogelator largely influences the properties of the resulting oleogel, including its firmness, stability, and viscoelastic behavior [5].

Oleogelators are generally classified into low-molecular-weight oleogelators (LMWOs) and high-molecular-weight oleogelators (HMWOs) [4]. HMWOs include polysaccharides, proteins, and certain polymers, while LMWOs commonly consist of fatty acids, waxes, lecithin, monoglycerides, and phytosterols [6]. Most LMWOs are hydrophobic and dissolve readily in oil, thus facilitating gel formation. In contrast, HMWOs exhibit polymeric characteristics that may improve viscoelastic stability to the gel network [7].

Among LMWOs, natural waxes are particularly attractive due to their ability to form gels at relatively low concentrations and their thermo-reversible behavior. They are typically composed of fatty acids and fatty alcohols, which allow them to form stable oleogels at concentrations as low as 1–4% (*w*/*w*) of the oil phase [8]. The melting points of natural waxes are generally moderately high, ranging between 50 °C and 80 °C, which give them the ability to form solid-like structures at room temperature, similar to conventional solid fats [9]. However, the downside of oleogels structured solely with natural waxes is that they may sometimes produce a coarse or waxy mouthfeel owing to incomplete melting under physiological conditions, which can negatively influence flavor perception and overall sensory quality [10].

To overcome these limitations, previous studies explored synergistic combinations of oleogelators to improve gel functionality and sensory characteristics [10,11]. Synergism refers to the interaction between two or more oleogelators that enhances gel strength, stability, or functionality beyond what each component can achieve individually by modifying the crystal size, packing density, and network organization. The interaction reduces the perception of coarse waxy textures while maintaining desirable structural properties. For example, Winkler-Moser et al. (2023) demonstrated that mixed wax oleogels containing BW, CW, and sunflower wax showed enhanced hardness and elasticity without adversely affecting the melting behavior [10]. In this study, rice bran oil (RBO)-based oleogels structured with beeswax (BW) and carnauba wax (CW) were developed and characterized to determine their physicochemical properties and to examine the synergistic effects of the two waxes as a potential alternative to commercial margarine. Furthermore, the oxidative stability and fatty acid profile of the formulated oleogels were analyzed and compared with those of commercial margarine as the control.

Both BW and CW have received considerable attention due to their complementary properties. BW is known for its strong gelling ability, with reports indicating effective oil gelation at concentrations as low as 1–3% (*w*/*w*), largely attributed to its needle-like crystal morphology [12]. It also exhibits hydrophobic, antioxidant, and favorable viscoelastic properties that contribute to the formation of stable and homogeneous gels [13]. Conversely, CW has one of the highest melting points among natural waxes (65–89 °C) and generally requires higher concentrations to achieve effective gelation, partly due to its spherulitic crystal morphology, which results in limited crystal formation at low concentrations [13].

In addition to the oleogelator, the choice of oil plays a crucial role in determining the physicochemical and visual properties of oleogels. Factors such as fatty acid composition, raw material characteristics, and processing conditions of the oil used can influence gel formation and stability. The type of oil can also affect the color and surface appearance of the final product [14]. While most studies have focused on commonly used vegetable oils such as soybean, sunflower, canola, and palm oil, RBO represents a promising alternative. Although it is not a common household oil, RBO contains approximately 47% monounsaturated, 33% polyunsaturated, and 20% saturated fatty acids, and it is rich in bioactive compounds with antioxidant and anti-inflammatory properties [15]. Its favorable fatty acid profile makes it suitable for developing oleogels intended to replace conventional solid fats while reducing the saturated fat content and eliminating trans fats.

Although BW- and CW-based oleogel systems have been studied extensively, the combined interactions in an RBO system remains limited, particularly the structure-function relationships that govern gel networking development and physicochemical behavior. Therefore, this study aimed to develop and characterize hybrid oleogels formulated using BW and CW as structuring agents in RBO. Specifically, the study sought to evaluate the synergistic effects of these waxes in altering the crystal morphology, thermal transitions, and viscoelastic network in RBO-based oleogels to produce a stable semi-solid fat alternative.

## 2. Results and Discussion

### 2.1. Thermal Behavior Profiles of RBO Oleogels

**Figure 1:**
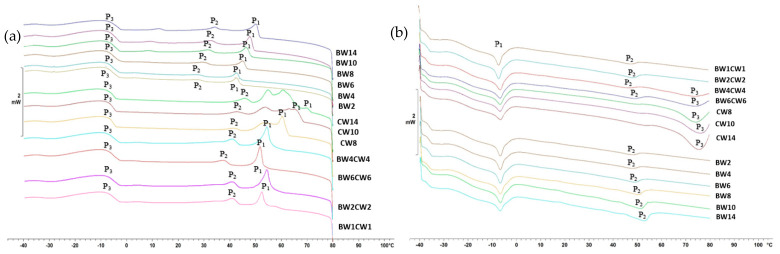
(**a**) Crystallization profile and (**b**) melting profile of natural waxes—incorporating RBO-based oleogels.

The thermal properties of the wax-structured oleogels were analyzed by using differential scanning calorimetry (DSC), and the crystallization and melting thermograms are presented in Figure 1. Three exothermic peaks were observed in the crystallization curves for all samples, whereas two or three peaks were detected in the melting curves. Oleogels structured with the same wax type showed similar thermal behavior patterns. The crystallization enthalpy (ΔH_c_) calculated from the peak area reflects the extent of crystal formation, where higher values indicate greater crystallization and may correlate with other properties such as oil binding capacity (OBC) and oleogel microstructure [16].

The crystallization behavior is strongly influenced by the composition of BW and CW. Wax esters, which account for approximately 71% of BW and 80–85% of CW, primarily determine the position of the initial crystallization peak [17,18]. In the study conducted by Pang et al., 2020, it is reported that oleogels containing CW exhibited higher onset crystallization temperatures compared with BW-structured oleogels [19]. In addition, decreasing wax concentration shifted the first crystallization peak toward lower temperatures, indicating delayed crystallization due to reduced wax ester content. Conversely, increasing CW concentration from 8% to 14% *w*/*w* increased the onset temperature from 62.43 °C to 71.38 °C. Similar trends were observed for BW and mixed-wax oleogels. The third crystallization peak, which remained nearly constant across all samples, was attributed to the crystallization of highly unsaturated triacylglycerols (TAGs) from RBO, which served as the continuous phase.

The first melting peak, P_1_, was similar across all samples and is associated with the melting of unsaturated TAGs in RBO in between −6.79 °C and −6.37 °C. As the temperature increased, additional peaks appeared corresponding to the melting of wax crystals. BW-structured oleogels exhibited peaks 1 and 2, whereas CW oleogels showed peaks 1 and 3. Mixed-wax oleogels displayed all three peaks, reflecting the combined melting behavior of BW and CW.

Increasing wax concentration also shifted the melting peaks toward higher temperatures, consistent with previous studies reporting that higher wax content increases the oleogel melting temperature [20]. Furthermore, the type of wax significantly influenced the melting behavior. BW typically melts between 61–65 °C, whereas CW has a higher melting range of approximately 82–86 °C [21,22]. Consequently, CW-structured oleogels exhibited higher melting temperatures (66.44–76.42 °C) compared with BW oleogels (48.20–53.03 °C), while mixed-wax oleogels displayed intermediate melting behavior (Table 1 and Table 2). Overall, the DSC thermograms demonstrate that both wax type and concentration determine the crystallization and melting behavior of the oleogel systems.

### 2.2. Microscopic Analysis

**Figure 2:**
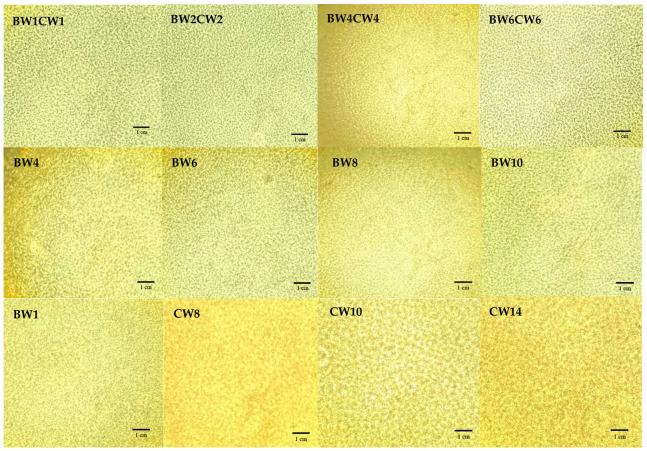
Microstructure of the oleogel crystals in the prepared oleogel samples observed under 40× magnification (1 cm represents 25 μm).

Microscopic analysis showed distinct crystal morphologies in oleogel systems structured using CW, BW, and their combination (Figure 2). BW-structured RBO oleogels predominantly formed needle-like crystals, which is consistent with the findings of BW-structured peanut oil systems by Zbikowska et al. (2022), whereas CW-structured RBO oleogels formed spherulitic crystals that seemed to overlap [23]. Based on previous studies carried out on CW-based olive oil oleogels, these structures are associated with the formation of rigid oleogel networks even though such systems require higher wax concentrations due to the relatively small size of the crystals [24]. Increasing the wax concentration produced a more compact crystal network for both BW- and CW-based oleogels. BW oleogels showed more densely packed needle-like crystals, while CW oleogels displayed greater overlapping of spherulitic crystals. Similar observations were reported by Kamali et al. (2019), who noted that higher wax concentrations promote crystal repositioning and changes in the microstructure [25].

For hybrid-wax oleogels, CW crystals dominated the microstructure although their size was smaller than those observed in single CW oleogels (8–14%). This may be due to the lower CW concentration, which increased the distance between crystals and reduced spherulitic overlap. Despite the lower CW content, oleogel formation was still achieved, suggesting a synergistic interaction between BW and CW. The needle-like crystals of BW may act as bridges between neighboring CW spherulites, forming a continuous network that structures the oil phase, which was particularly evident in the BW4CW4 and BW6CW6 oleogel systems (Figure 2).

### 2.3. Rheological Analysis

#### 2.3.1. Frequency Sweep Test

**Figure 3:**
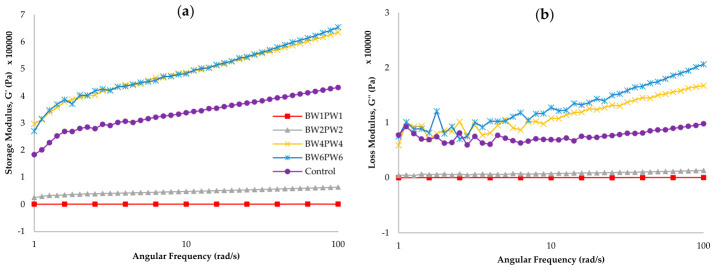

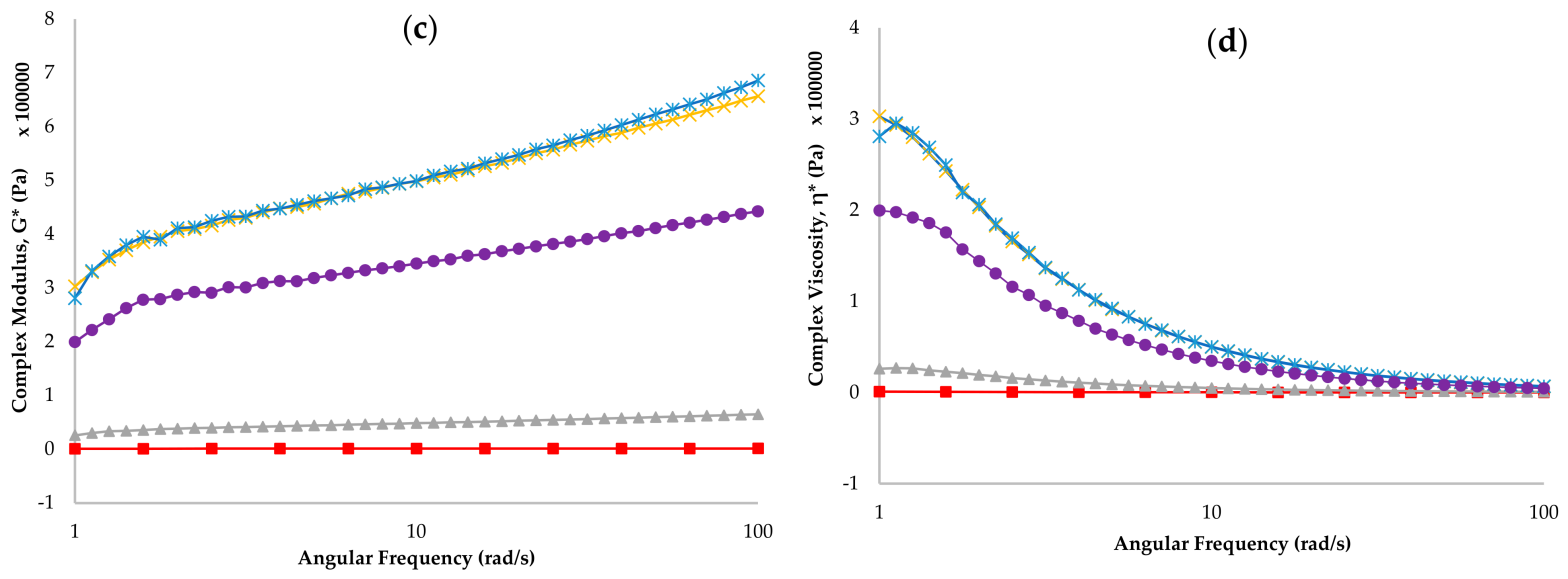
Changes in the (**a**) storage modulus, (**b**) loss modulus, (**c**) complex modulus, and (**d**) complex viscosity of the hybrid wax-based RBO oleogels over angular frequency.

**Figure 4:**
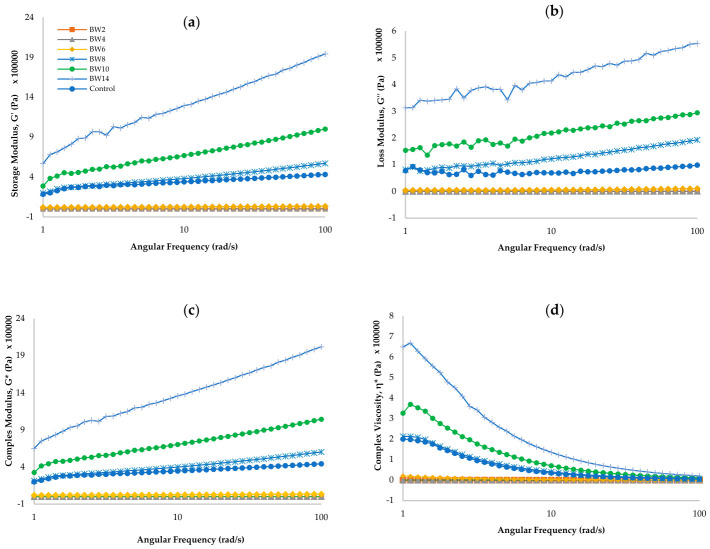
Changes in the (**a**) storage modulus, (**b**) loss modulus, (**c**) complex modulus, and (**d**) complex viscosity of the BW-based RBO oleogels over angular frequency.

**Figure 5:**
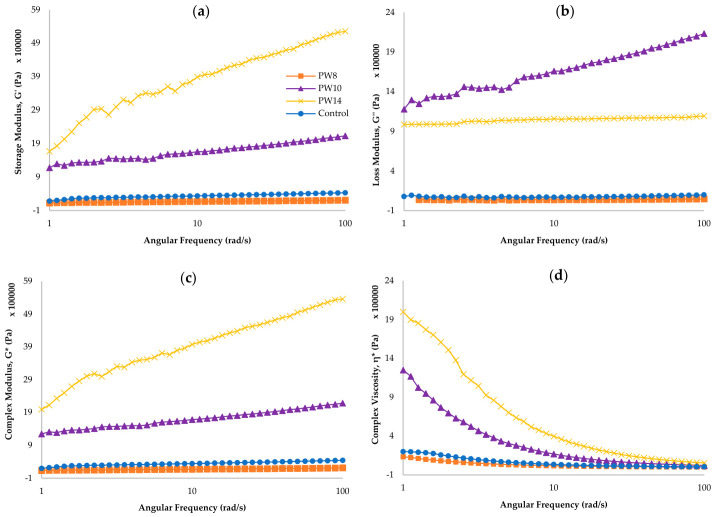
Changes in the (**a**) storage modulus, (**b**) loss modulus, (**c**) complex modulus, and (**d**) complex viscosity of the CW-based RBO oleogels over angular frequency.

A frequency sweep test was conducted within the linear viscoelastic region (LVR) to evaluate the viscoelastic behavior of the oleogels without disrupting their structure [26]. Our results highlighted that all samples exhibited G′ values higher than G″ across the tested frequency range, indicating the typical elastic behavior of gel-like systems (Figure 3, Figure 4 and Figure 5). Figure 4 and Figure 5 showed a steeper increase in G′ with increasing angular frequency for CW14 and BW14. This is because weakly structured three-dimensional networks are more sensitive to deformation, particularly when excessive crystal aggregation reduces effective crystal packing and interconnectivity [27]. Chai et al. (2022) also reported that higher BW concentrations can produce more brittle oleogels with greater frequency dependence [28]. The remaining samples showed a gradual increase in G′, indicating typical gel-like behavior [29]. Complex viscosity decreased with increasing angular frequency, indicating shear-thinning behavior. BW2 and BW4 showed the lowest flow resistance among BW oleogels, while BW1CW1 showed the lowest among hybrid systems. A synergistic effect was observed in BW2CW2 and BW6CW6, which showed higher resistance to flow than BW oleogels at the same concentration. The coexistence of both fibrillar needle crystals (BW) and space-filling spherulites (CW) increased crystal connectivity and hierarchical network formation, leading to enhanced mechanical strength of the oleogel systems [30]. Overall, BW oleogels with concentrations above 6% and CW-based oleogels exhibited higher resistance to flow, likely due to stronger crystal network formation and interactions within the oleogel structure, as evidenced in Figure 2.

#### 2.3.2. Dynamic Temperature Ramp

**Figure 6:**
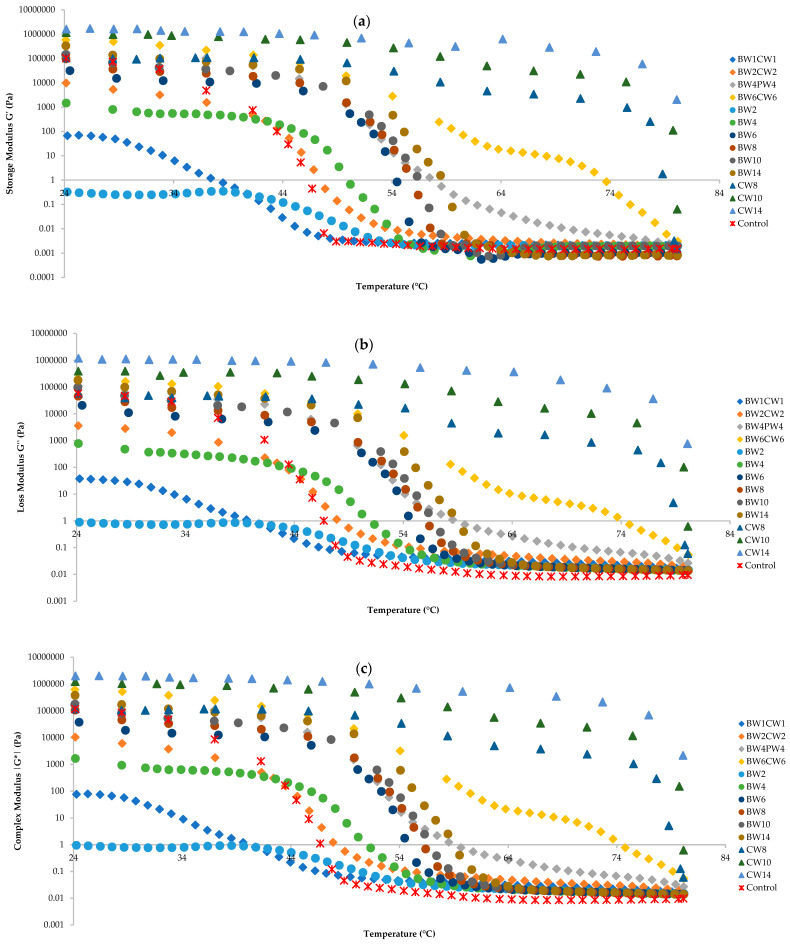
Changes in the (**a**) storage modulus, (**b**) loss modulus, and (**c**) complex modulus of the RBO-based oleogels structured with different concentrations and combinations of waxes over temperature.

Dynamic temperature ramp analysis showed that G′, G″, and G* decreased with increasing temperature (Figure 6). BW2 showed prominent viscous properties even at low temperatures, suggesting that the gelation occurred below the critical concentration. This behavior could be associated with the predominance of crystalline spherulites with lower structuring ability, which leads to less homogeneous crystal organization and a weaker gel network at low BW concentration [28,31]. Most samples showed a sharp decline in G′ at higher temperatures, which was characterized by the reduced elastic properties and the increased viscous properties. During the melting transition of the oleogels, a crossover point occurs where the curve for G′ intersects with that of G″ and the value for G″ dominates from that point [32]. The temperature at which this decline occurred was consistent with the melting temperatures observed in the DSC thermograms (Table 2), which is similar to findings reported by Martins et al. (2016) [31]. For example, BW14 showed a melting temperature of 53.03 °C. By plotting this value into the G′ curve for BW14 in Figure 6, this melting temperature lies close to the sharp turning point which resembles a 90° angle. The complex modulus (G*) reflects the overall resistance of the samples to temperature-induced deformation. As shown in Figure 6, oleogels with higher wax concentrations showed greater resistance to deformation owing to the formation of a more densely packed and interconnected crystal network. In addition, CW-containing oleogels generally showed higher resistance due to the intrinsically higher melting point, stiffness, and rigidity of CW [33].

#### 2.3.3. Temperature Sweep Test

**Figure 7:**
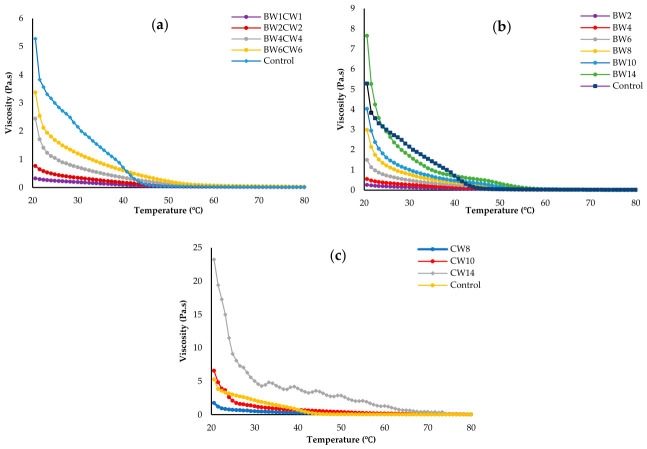
Changes in viscosity of the (**a**) hybrid wax-based, (**b**) BW-based, and (**c**) CW-based oleogels structured over temperature.

With reference to Figure 7, the viscosity of all oleogel samples decreased with increasing temperature. This behavior is associated with a change in state particularly for semi-solid and solid oleogel samples or known as the gel–sol transition as the temperature increases which can be explained based on the kinetic particle theory [34]. The kinetics of oleogels are important in food applications since phase transitions can influence desirable organoleptic properties [34]. As the temperature increases from 20 to 80 °C, oil and oleogelator molecules gain kinetic energy, causing faster molecular motion and weakening the intermolecular interactions that maintain the gel structure [35]. The semi-solid structure may first transition into a liquid crystal phase and eventually into a fully liquid state once the melting point of the oil and oleogelator is exceeded [36]. This transition increases the flow behavior of RBO oleogels as the temperature rises, as indicated by the decrease in viscosity. The type and concentration of wax influenced the viscosity at different temperatures, consistent with their thermal behavior observed in DSC analysis. Among the samples, CW14 showed the highest viscosity and required a higher temperature to reach a similar consistency to the other oleogels, likely due to the higher melting point of CW compared with BW. In addition, a high concentration of CW forms more ordered and tightly packed crystalline domains with stronger crystal–crystal interactions which collectively contribute to increased viscosity and thermal resistance.

### 2.4. Viscosity

**Figure 8:**
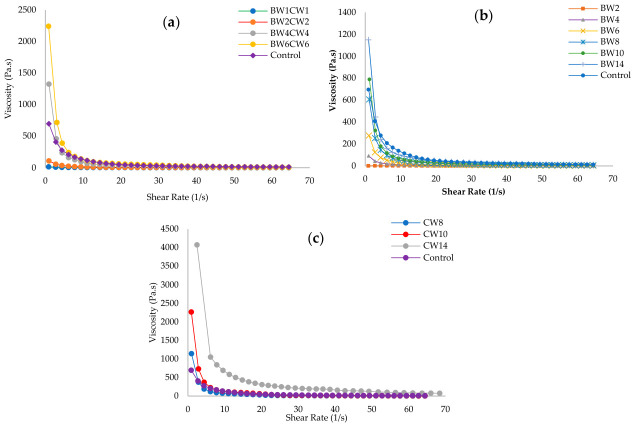
Changes in viscosity in (**a**) hybrid wax-based, (**b**) BW-based, and (**c**) CW-based RBO oleogels over shear rate.

Based on Figure 8, all oleogel samples showed shear-thinning behavior as the viscosity decreased with increasing shear rate. Similar behavior was reported in oleogels by Espert et al. (2022) [37]. This can be explained by the disruption of the hydrogen bonding and Van der Waals forces that maintain the oleogel network as the shearing forces increase [37,38]. Our results revealed that an increase in wax concentration corresponded to greater resistance to shear due to the formation of stronger crystalline networks, in agreement with Kwon and Chang (2022) who observed similar trends [38]. The resistance of the oleogel samples to shear can also be explained by their textural properties, in particular their hardness (Table 3). In BW-structured oleogels, increasing the BW concentration increased the hardness and shear resistance. This behavior was further supported by the increase in the consistency index, a rheological parameter that reflects the apparent viscosity and flow resistance of the material. We observed an approximately eightfold increase in the consistency index as the BW concentration increased from 4% to 14%, indicating the formation of a stronger oleogel network at higher BW concentrations. Interestingly, our findings indicated that the mixed-wax oleogel systems exhibited higher viscosity than the oleogels structured with either wax alone at the same total concentration, suggesting a synergistic interaction between the two waxes that forms a stronger crystalline network.

### 2.5. Texture Profile Analysis

Ten out of the 13 oleogel samples formed gel successfully. The remaining three were classified as “take liquid” due to their runny consistency and could not be analyzed for textural properties. Preliminary studies indicated that CW-based RBO oleogels require higher CW concentrations to form gels because of the limited crystal formation at low concentrations and the nature of the CW crystal morphology. In contrast, BW can structure RBO at lower concentrations, with a reported threshold of 3% *w*/*w*, which explains why BW2 failed to form a gel. Texture profile analysis (TPA) was performed on the 10 gelled samples to measure hardness, adhesiveness, and cohesiveness, simulating the compressive action of chewing.

Hardness, which measures resistance to deformation, generally increased with higher wax concentration (Table 3). However, BW–CW hybrid oleogels (BW4CW4 and BW6CW6) exhibited hardness similar to 14% CW oleogel and the control (*p* > 0.05). BW-based oleogels had comparable hardness to lower-concentration CW samples (8–10%), indicating that CW largely contributes to stiffness in RBO oleogels. Among the BW-based samples, BW10 was significantly harder than BW4 and BW6, which were the two lowest concentrations of the BW-based oleogels. The unexpectedly lower hardness of BW14 may be due to high deformation during the first compression and poor recovery, also known as resilience, after the first compression. Our results suggested that spherulitic crystals (CW) tend to build a more interconnected and space-filling network with stronger contact points between crystals which makes the gel harder. Although needle-like crystals (BW) can form a gel at lower concentrations, the network is more loosely connected, which therefore leads to a weaker gel structure.

Adhesiveness measures the force needed to detach the probe after compression [39]. According to Table 3, no significant difference (*p* > 0.05) was observed among BW10, BW4CW4 and the control, indicating their adhesiveness closely resembled the control. Cohesiveness reflects a sample’s ability to resist deformation until structural failure, which is dependent on internal bonding [40]. Significant differences (*p* < 0.05) from the control were observed in BW4, BW6CW6, CW8, and CW14. The control exhibited unusually high cohesiveness despite high value in hardness, which is likely due to strong hydrogen bonds among the fat crystals. Among samples that are not significantly different (*p* > 0.05) from the control, the 10% BW oleogel closely resembled the control and showed high values in both hardness and cohesiveness.

### 2.6. Oil Binding Capacity

The gelation capability of natural wax is a critical property of wax-structured oleogels as it reflects the efficiency of gel formation and affects their applications in food systems. The structure of oleogels is determined by the crystal network formed by the wax and the extent of oil immobilization depends on the strength of the hydrogen bonding and other intermolecular interactions between the oil and the oleogelator. Barroso et al. (2022) reported that the cooling rate of the oil–gelator mixture can influence the oil binding capacity (OBC) of the oleogel system since rapid cooling tends to produce smaller and uniform crystals which will form a more compact and rigid structure, whereas slow cooling may produce larger and uneven crystals [41].

According to Table 4, the OBC values were not significantly different (*p* > 0.05) among the RBO oleogel samples, except for BW1CW1. This suggests that the synergistic effect of BW and CW may only occur above a critical wax concentration of 1% for each wax. Despite statistical differences (*p* < 0.05) in OBC between BW1CW1 and BW2 or BW2CW2, their visual appearances were similar. Environmental temperature may also influence the OBC measurements, as all samples were frozen overnight before centrifugation. During storage at room temperature, BW1CW1, BW2CW2, and BW2 were liquid. After removal from the freezer, BW1CW1 showed signs of melting, indicating insufficient wax to effectively entrap the oil and thus a higher proportion of unbound oil molecules. Nonetheless, the calculated OBC values were high, demonstrating strong interactions between the oleogelators and RBO. Overall, BW appeared more efficient than CW in entrapping RBO due to nearly ideal OBC values in all BW-based samples.

### 2.7. Slip Melting Point

For the RBO oleogels, the slip melting point (SMP) was measured as the temperature at which the sample column in a capillary tube begins to rise due to hydrostatic pressure. Table 5 shows that the SMP generally increased with wax concentration. Higher wax content produces a more compact crystal network with stronger hydrogen bonds and Van der Waals forces. Moreover, a higher amount of oleogelator is able to entrap more oil molecules, which produces RBO oleogels with higher rigidity at higher wax concentration. Thus, a higher temperature is required. Significant differences (*p* < 0.05) were observed for BW4 and BW14 oleogels, while BW-based oleogels above 4% and BW–CW hybrids above 2% BW–2% CW showed similar SMPs. For CW-only oleogels (8–14%), each concentration displayed a distinct SMP. Notably, hybrid BW–CW oleogels had higher SMPs than BW alone at the same concentration, demonstrating the synergistic effects between BW and CW. BW allows gelation at lower CW concentrations, while CW increases the melting point of BW which innately has a low melting point.

### 2.8. Oxidative Stability Analysis

The RBO oleogel samples were incubated at 60 °C in a dim environment to accelerate oxidation, and peroxide values (PV) were measured every four days over 20 days to monitor oxidative stability. Peroxides are primary lipid oxidation products that are typically broken down as the degradation process is increased at high temperatures [42]. As a result, the PV measurements near the end of incubation may not accurately reflect the extent of lipid oxidation since the primary peroxides have been converted into secondary oxidation products. Table 6 shows no significant difference (*p* > 0.05) in PV on days 16 and 20, while significant differences (*p* < 0.05) occurred on days 4, 8, and 12. This indicates that most primary peroxides broke down after day 12 of incubation, which led to a subsequent decline in PV measured from day 12 to day 16.

Increasing BW concentration generally improved oxidative stability, with lower PVs observed on days 4, 8, and 12. An exception was BW8, which showed the lowest stability among the BW-based oleogels. For hybrid oleogels, CW improved the oxidative stability of the BW-based samples. This is evident from Table 6 which shows BW1CW1 having a lower PV than BW2 on days 4, 8, and 12. Similarly, BW2CW2 and BW4CW4 demonstrated a lower PV than BW4 and BW8, respectively, during the accelerated storage conditions. These findings suggested that the combination of BW and CW enhanced the resistance towards lipid oxidation more effectively than BW alone at an equivalent total wax concentration. Moreover, our study also showed that BW6CW6 exhibited a very low PV compared to other samples, only increasing by 12 mEq/kg after 20 days of storage. The enhanced stability may be attributed to the formation of a more compact and stable oleogel network which improves the oxidative stability of oil by immobilizing the liquid oil phase within the three-dimensional crystal network, thereby limiting the diffusion of oxygen and reducing the mobility of pro-oxidants. Overall, these results indicate that increasing wax concentration and optimizing the BW–CW combinations could retard lipid oxidation in RBO-based oleogels by promoting a stronger and denser crystal network that protects RBO from oxidation.

CW-based oleogels showed no clear trend dependent on concentration but showed a high stability overall, with PV below 20 up to day 12. This is likely due to the high content of saturated fatty acid esters in CW, derived from *Copernicia prunifera*, which enhances resistance to oxidation [43]. Since PV mainly reflects primary oxidation products, our present study provides an initial assessment of the oxidative rate of the oleogel system. Future studies incorporating other oxidative indices including acid value, iodine value, and *p*-anisidine value would provide a better understanding of lipid oxidation in RBO-based oleogels.

### 2.9. Colorimetric Analysis

Table 7 shows that all RBO oleogel samples exhibited lower L* values than the control, with BW14 being the lightest (69.56 ± 0.37) and BW4 the darkest (34.42 ± 1.04) among the oleogel samples. Our results showed that higher wax concentration was associated with higher lightness (L*) values and vice versa. For instance, among all the BW-based RBO oleogels, BW4 showed the lowest lightness among them, and this was similarly observed in CW-based RBO oleogels as well as the hybrid wax RBO oleogels. This suggests that increasing the wax concentration can make oleogels appear lighter (higher L*) due to the formation of a more uniform crystalline network of smaller and more numerous crystals. This phenomenon improves the light scattering and reduces localized light absorption, thereby leading to a brighter or whiter appearance.

For a* values, all oleogels were negative except BW1CW1 which had a positive value (0.04 ± 0.12), appearing slightly redder. Increasing the wax concentration generally increased greenness. The control sample, however, portrayed a higher degree of redness as opposed to the RBO oleogel samples. Among the RBO oleogels, BW6CW6 was the greenest, while BW1CW1 was the reddest. The observed greenness in BW- and CW-based oleogels aligns with previous studies [44,45]. The control sample appeared redder than all oleogels.

For b* values, all oleogels were more yellow than blue but less yellow than the control. Increasing the wax concentration tended to increase yellowness. Among all samples, CW14 was the most yellow (18.36 ± 1.09) and BW1CW1 the least yellow/bluest (6.44 ± 0.36). Overall, at low wax concentrations, the color of the oleogel system is largely determined by RBO which has the continuous phase, while at higher wax concentrations, the incorporated wax dominates the color of the resulting oleogel.

### 2.10. Fatty Acid Composition

RBO generally contains a high proportion of unsaturated fatty acids (~80%) and moderate amounts of saturated fatty acids (~20%) [46]. Since RBO is largely unsaturated, the oxidative stability of RBO-based oleogels depends on factors such as OBC and the oleogel crystal network. Table 8 shows the fatty acids found in the samples in order of chain length, with lauric acid (C12:0), myristic acid (C14:0), palmitic acid (C16:0), stearic acid (C18:0), oleic acid (C18:1-cis n9), vaccenic acid (C18:1-trans-11), linoleic acid (C18:2-cis n6), α-linolenic acid (C18:3 n3), and arachidonic acid (C20:4 n6).

The fatty acid compositions of the RBO oleogels closely resembled that of RBO since RBO was used as the continuous phase. Among the samples, BW2 had the highest resemblance to RBO, which is likely due to the fact that BW was added in very minute concentration. Apart from a small amount of myristic acid at 0.43%, all the other fatty acids were identical to RBO. Compared to the control sample (margarine), the oleogel samples contained lower amounts of saturated fatty acids. The control had significantly higher levels of myristic, palmitic, and stearic acids, with palmitic and stearic nearly double those in most oleogels. The control sample also had much lower amounts of monounsaturated and polyunsaturated fatty acids, such as oleic, linoleic, linolenic, and arachidonic acids. Lauric acid (~4.94%) was detected only in the control. The fatty acid profile of RBO was consistent with the previous report [47]. Variations in unsaturated fatty acids among samples may be due to storage effects or the type of wax used [31,48]. Overall, RBO-based oleogels structured with BW, CW, or their combinations have a healthier lipid profile than commercial margarine, which supports that they are healthier alternatives.

## 3. Conclusions

This study investigated the physicochemical properties of 13 RBO-based oleogel samples and used margarine as a control. The type and concentration of the waxes were the main factors influencing the measured properties. Hybrid BW–CW oleogels exhibited synergistic effects in some properties, particularly in thermal behavior. When BW was used alone, the melting point was lower, but when CW was used together with BW, the melting point of the hybrid oleogels increased. BW facilitated gelation at low concentrations of both waxes, which CW alone could not achieve. In terms of oxidative stability, RBO oleogels had lower stability than the control due to a higher unsaturated fatty acid content whereas the control showed greater stability because of its higher saturated fatty acid content. Among the samples, BW4CW4 and BW6CW6 showed potential for further study to explore the physiochemical properties that were not explored in this study. Although CW10 and BW10 also demonstrated favorable physicochemical properties, the relatively high wax concentration required may limit their practical application and cost-effectiveness in food formulations. Overall, RBO-based oleogels demonstrate promise as healthier alternatives, although improvements in stability and further optimization are needed. To support this optimization, future work should employ advanced structural techniques (e.g., X-ray diffraction (XRD), small-angle and wide-angle X-ray scattering (SAXS/WAXS), cryo-scanning electron microscopy (cryo-SEM) or confocal microscopy) to better understand the structure–property relationships of RBO oleogel systems.

## 4. Materials and Methods

### 4.1. Materials

Refined RBO (King Brand, Bangkok, Thailand) and commercial margarine were purchased from Lotus’ supermarket, where the former was used as the organic oil phase and the latter was used as the reference sample throughout the study. Based on the product label, the commercial margarine contained 82.4% total fat, with no reported protein, carbohydrate, or dietary fiber. The main ingredients included palm-based oil and sunflower oil, salt, emulsifiers, flavoring substances, vitamins, antioxidants, coloring substances, and stabilizer. Food grade CW and BW were acquired from Shopee store (Take it Global Sdn. Bhd., Sungai Jawi, Malaysia) to be used as the oleogelators. All chemicals and solvents used were of analytical grade.

### 4.2. Preparation of Oleogel Samples

A total of thirteen different oleogel systems were prepared, each with a total weight of 100 g. The formulations for each system varied in the concentration of BW, or in combinations of BW with CW, and CW with RBO. The BW concentrations ranged from 2–14% due to its strong gelation ability at low concentrations, whereas the CW concentrations ranged from 8–14% because our preliminary study showed that concentrations below 8% did not form stable oleogels. Therefore, the required amounts of RBO and the oleogelators were weighed separately based on the specified percentage (*w*/*w*) composition of the oleogel sample, as presented in Table 9. The 1:1 binary wax combinations were selected to enable direct comparison between single-wax and hybrid-wax systems at equivalent total wax concentrations [49].

The RBO was first heated under continuous stirring. Once a temperature of 80 °C was reached, the pre-weighed CW and BW were added to the heated RBO slowly, either in combination or separately. Then, the mixture was stirred until the waxes were fully dissolved, forming a clear and homogeneous solution. After complete dissolution, without heating, the mixture was stirred manually with a spatula for an additional 3 min. The hot mixture was then poured into labelled, microwavable plastic containers and left to cool naturally to ambient temperature (25 ± 3 °C) at an approximate rate of 3 °C/min. The samples were then maintained at ambient conditions for 24 h to allow complete gelation. The formed oleogels were stored under these conditions until further analysis was carried out.

### 4.3. Determination of Physiochemical Properties of Oleogel Samples

#### 4.3.1. Thermal Profile Analysis

The thermal behavior of the oleogel samples was investigated using a differential scanning calorimeter (DSC) (Model DSC823e, Mettler Toledo, Greifensee, Switzerland). Approximately 5 mg of each sample was weighed into an aluminum crucible and then hermetically sealed. An empty sealed pan was used as the reference. The DSC was programmed to heat the samples from 25 °C to 80 °C and they were held for 5 min to ensure complete melting of all crystal nuclei. They were then cooled to −40 °C at a rate of 5 °C/min and maintained at this temperature for 5 min before being reheated to 80 °C at the same rate. Crystallization and melting transitions curves were obtained from the resulting thermograms generated for each oleogel sample. Key thermal parameters, including transition temperature, crystallization onset temperature (T_oc_), crystallization offset temperature (T_fc_), crystallization enthalpy (ΔH_c_), and peak temperature (T_p_), were determined using STARe Excellence Thermal Analysis Software version 9.

#### 4.3.2. Microscopic Study

The oleogel samples were first melted on a hotplate. A thin layer of the melted oleogel was then spread onto preheated microscopic slides and carefully covered using a coverslip. After recrystallization, the microstructures were then observed under an inverted microscope (Model ECLIPSE Ti2, Nikon, Tokyo, Japan) at 40× magnification to evaluate the crystal structure formed [50].

#### 4.3.3. Rheological Analysis

The rheological properties of the oleogel samples were evaluated using a Discovery Hybrid Rheometer (Model DHR-1, TA Instruments, New Castle, DE, USA) in accordance with the method described by Kwon and Chang (2022) with modifications [38]. Briefly, the oleogel samples were carefully loaded onto the Peltier plate and the rheometer head was lowered slowly to compress the sample until reaching a gap distance of 1 mm. Any excess sample was carefully scraped off using a plastic spoon, and after each test, the oleogel samples were cleaned off from both the Peltier plate and the 40 mm parallel plate, which were then washed with isopropanol before loading the next sample. All analyses were performed in triplicate.

##### Shear Viscosity Test

Shear viscosity was measured at 20 °C by increasing the shear rate from 0 to 100 s^−1^. The corresponding shear stress versus shear rate curves were recorded for data analysis.

##### Temperature Sweep Test

The analysis was conducted by heating the samples from 20 °C to 80 °C at a rate of 5 °C/min while maintaining a constant shear rate of 100 s^−1^. The graph of viscosity vs. temperature was then recorded for all samples for subsequent data analysis.

##### Frequency Sweep Test

The analysis was carried out at a strain of 0.1% which is within the linear viscoelastic limit. The angular frequency varied from 1 Hz to 100 Hz at constant temperature of 20 °C. The graph of storage modulus (G′), loss modulus (G″), and complex modulus (G*) vs. angular frequency was then obtained for all samples for subsequent data analysis.

##### Dynamic Temperature Ramp

Dynamic temperature ramp analysis was performed using a fixed strain of 0.1% and an angular frequency of 1 Hz. During the analysis, the temperature was increased from 20 °C to 8 °C at a heating rate of 5 °C/min. The graph of storage modulus (G′), loss modulus (G″), and complex modulus (G*) vs. temperature was then obtained for all samples for subsequent data analysis.

#### 4.3.4. Texture Analysis

Semi-solid and solid oleogel samples were evaluated for hardness, adhesiveness, and cohesiveness using a texture analyzer (Model TA.XT plus, Stable Micro System, Surrey, UK) equipped with a 35 mm cylindrical probe. The samples were shaped into 2 cm × 2 cm × 2 cm cubes and placed on the sample loading platform prior to compression testing. Prior to measurement, the height and force of the instrument was calibrated using a 5 kg calibration weight. Each sample underwent two consecutive compression cycles to a penetration depth of 12 mm, with both the downward and return movements of the probe carried out at a constant speed of 2 mm/s. The textural parameters were recorded for each sample.

#### 4.3.5. Oil Binding Capacity

The OBC analysis was conducted following the method of Öğütcü and Yılmaz (2015) with slight modifications [45]. The samples were first melted and 1 mL of each melted oleogel was transferred into a 15 mL Eppendorf conical tube before being stored in the refrigerator for 1 h. The tubes were then weighed, and the initial weight of each tube was recorded. After that, the tubes were centrifuged at 10,000 rpm for 15 min to release the liquid oil. The released oil was carefully decanted by inverting the tubes onto absorbent paper, and the tubes were weighed again, and the final weight of the tubes was recorded. The OBC values were determined gravimetrically based on the initial and final weights using Equation (1).

Mass of released oil (g) = Initial weight of tube (g) − Final weight of tube (g)

% Oil released = (Mass of released oil (g))/(Total mass of sample (g)) × 100%

OBC = 100 − % Oil released(1)

#### 4.3.6. Slip Melting Point

The SMP of the samples was determined using the capillary tube method [51]. The melted oleogels were filled into capillary tubes up to the 1 cm mark and stored in a freezer for 24 h. After storage, each capillary tube was attached to the bottom tip of a glass thermometer using a rubber band. The thermometer was clamped to a retort stand to ensure proper positioning, while the lower end of the thermometer was placed in the beaker filled with an ice-water bath placed on a hotplate. The water bath was gradually heated, and the temperature at which the oleogel column began to rise was recorded as the SMP.

#### 4.3.7. Oxidative Stability Test

An accelerated storage test was conducted at 60 °C for 20 days to investigate the oxidative stability of the oleogel samples, based on the method reported by Wang et al. (2024) with minor adjustments [22]. The peroxide value (PV) was analyzed every 4 days by iodometric titration. Briefly, an acetic acid–chloroform solution (3:2, *v*/*v*) was prepared by mixing glacial acetic acid and chloroform. Approximately 5 g of sample was dissolved in 30 mL of the prepared solvent mixture, followed by the addition of 1 mL of saturated potassium iodide solution and swirling for 1 min. This was followed by the addition of 30 mL of distilled water with thorough mixing, and finally 0.5 mL of 1% starch solution was added as an indicator. The liberated iodine was titrated with 0.01 N sodium thiosulfate solution until the endpoint was reached, indicated by a color change from blue-green to milky white. The PV was then determined using Equation (2):

Peroxide Value = (V × N × 1000)/Ws(2)

where

Peroxide Value = milliequivalents (mEq) peroxide per kg of sample

Ws is the sample weight in grams

N is the normality of sodium thiosulfate (Na_2_S_2_O_3_) solution

V is the volume of sodium thiosulfate (Na_2_S_2_O_3_) solution used

#### 4.3.8. Colorimetry

The instrument was calibrated and zeroed using a standard white calibration plate prior to measurement. Liquid and semi-solid oleogel samples were poured into mini petri dishes, while solid samples were measured directly from a flat and even surface. Color parameters L* (lightness), a* (greenness to redness), and b* (blueness to yellowness) were recorded using a CR-400 colorimeter (Konica Minolta, Tokyo, Japan).

#### 4.3.9. Fatty Acid Content

The fatty acid profile was determined in accordance with our previous study [52]. Approximately 100 mg (± 5 mg) of oleogel sample was added with 5 mL of hexane in a centrifuge tube and the mixture was vortexed for 1 min. Subsequently, 250 μL of 0.5 M sodium methoxide was added and the mixture was vortexed for 1 min while pausing every 10 s to allow the vortex to collapse. After this, 5 mL of saturated sodium chloride solution was added. The mixture was shaken vigorously for 15 s and allowed to stand for 10 min to facilitate phase separation. After 10 min, 2 mL of the upper hexane layer was removed and transferred into a vial containing 2 mL of hexane solution to dilute the methyl esters. The vial was capped and inverted to mix the contained mixture. The resulting mixture was then used for subsequent GC analysis.

The fatty acid composition of oleogel samples was analyzed using a gas chromatograph (GC) (Model GC-2010, Shimadzu Corporation, Kyoto, Japan) equipped with a flame ionization detector (FID) and a BPX 70 column (60 m × 0.25 mm × 0.25 μm). The injector temperature was set at 220 °C, while the FID temperature was maintained at 250 °C. Helium and nitrogen were used as the carrier and makeup gases, respectively, at flow rates of 23 mL/min and 20 mL/min. The initial column temperature was set at 115 °C and programmed to increase to 180 °C at a heating rate of 8 °C/min. Prior to injection, the microsyringe was rinsed three times with hexane and three times with the sample solution. A 2 μL aliquot of the derivatized sample was then injected into the injection port. After injection, the analysis was initiated immediately. The syringe was rinsed with hexane before being used for the next sample. The percentage of fatty acid (% FA) was calculated based on the peak area of each fatty acid. FAME standard mixture (Supelco, Bellefonte, PA, USA) containing 37 known compounds (C4:0–C24:0) was used as the reference.

### 4.4. Statistical Analysis

The data were analyzed using SPSS software (IBM SPSS Statistics version 26). Each formulation was prepared independently in duplicate and most analyses were performed in triplicate. Results were presented as mean ± standard deviation. One-way analysis of variance (ANOVA) was performed to compare the statistical significance among the samples. Tukey’s multiple comparison test was applied to identify significant differences between means at a 95% confidence level.

## Figures and Tables

**Table 1:** Crystallization onset (T_oc_), crystallization offset (T_fc_), crystallization enthalpy (∆H_c_) oleogel samples at 5 °C/min interval.

Samples	Transition Temperature (°C)			T_oc_ (°C)	T_fc_ (°C)	∆H_c_ (J/g)
	P_1_	P_2_	P_3_			
BW1CW1	53.01	36.46	−9.03	52.25	−26.44	10.56
BW2CW2	53.72	36.75	−9.16	52.60	−26.35	11.47
BW4CW4	51.79	37.95	−9.22	53.03	−26.52	12.95
BW6CW6	54.46	41.11	−9.22	55.99	−26.26	17.01
BW2	42.34	27.89	−9.02	43.11	−24.86	15.52
BW4	42.61	28.11	−8.81	44.25	−24.79	10.60
BW6	45.11	30.11	−9.22	46.52	−26.58	13.10
BW8	46.45	31.77	−8.56	48.27	−25.91	10.58
BW10	47.79	32.78	−8.88	49.25	−26.65	14.30
BW14	50.11	34.28	−8.56	51.50	−24.70	11.32
CW8	60.45	41.77	−8.89	62.43	−25.76	19.77
CW10	65.61	42.78	−8.89	68.36	−25.13	13.97
CW14	70.02	45.78	−9.89	71.38	−27.41	14.84

**Table 2:** Melting onset (T_om_), melting offset (T_fm_), and melting enthalpy (∆H_m_) of oleogel samples at 5 °C/min interval.

Samples	Transition Temperature (°C)			T_om_ (°C)	T_fm_ (°C)	∆H_m_ (J/g)
	P_1_	P_2_	P_3_			
BW1CW1	−6.51	45.98	-	−11.12	50.43	−11.67
BW2CW2	−6.47	46.12	-	−11.17	50.54	−12.34
BW4CW4	−6.46	46.37	73.86	−11.56	78.92	−14.57
BW6CW6	−6.53	49.21	74.53	−12.80	79.15	−17.19
BW2	−6.79	47.90	-	−10.01	50.44	−13.57
BW4	−6.79	48.20	-	−10.15	50.68	−13.68
BW6	−6.71	48.53	-	−10.18	51.58	−15.16
BW8	−6.70	50.20	-	−11.30	52.96	−16.41
BW10	−6.71	51.19	-	−11.36	53.77	−16.62
BW14	−6.78	53.03	-	−11.66	55.03	−14.58
CW8	−6.45	-	66.44	−12.18	79.52	−7.78
CW10	−6.45	-	74.60	−12.43	79.51	−12.42
CW14	−6.37	-	76.42	−12.60	79.92	−11.79

**Table 3:** Effect of the type of wax, concentration of wax and combination of waxes on the textural properties (hardness, adhesiveness, and cohesiveness) of RBO oleogels in comparison to a control sample (margarine).

Samples	Hardness (N)	Adhesiveness (mJ)	Cohesiveness
BW2	t.l	t.l	t.l
BW4	127.87 ^d^ ± 9.12	−106.46 ^a^ ± 0.49	0.552 ^a^ ± 0.003
BW6	159.69 ^d^ ± 17.86	−149.13 ^a^ ± 10.33	0.432 ^abc^ ± 0.057
BW8	249.02 ^cd^ ± 26.10	−243.80 ^a^ ± 55.77	0.372 ^bcde^ ± 0.075
BW10	369.58 ^bc^ ± 60.14	−277.20 ^ab^ ± 45.78	0.441 ^abc^ ± 0.004
BW14	277.00 ^cd^ ± 17.15	−222.86 ^a^ ± 18.52	0.460 ^ab^ ± 0.001
BW1CW1	t.l	t.l	t.l
BW2CW2	t.l	t.l	t.l
BW4CW4	558.89 ^a^ ± 28.93	−333.37 ^ab^ ± 1.35	0.277 ^def^ ± 0.011
BW6CW6	537.55 ^ab^ ± 35.66	−183.30 ^a^ ± 87.51	0.182 ^f^ ± 0.060
CW8	245.54 ^cd^ ± 62.85	−91.26 ^a^ ± 4.04	0.254 ^ef^ ± 0.020
CW10	250.76 ^cd^ ± 41.90	−145.44 ^a^ ± 20.73	0.310 ^cdef^ ± 0.001
CW14	560.33 ^a^ ± 102.36	−244.36 ^a^ ± 104.98	0.248 ^ef^ ± 0.021
Control	692.45 ^a^ ± 60.52	−701.38 ^b^ ± 10.04	0.409 ^bcd^ ± 0.011

t.l means take liquid. Units for hardness and adhesiveness are Newton (N) and mJ (millijoule), respectively whereas cohesiveness is unitless. Mean values with the same superscript are not significantly different and vice versa.

**Table 4:** Effect of the type of wax, concentration of wax, and combination of waxes on the OBC of RBO oleogels.

Samples	Oil Binding Capacity (%)
BW2	97.41 ^a^ ± 0.0167
BW4	99.44 ^a^ ± 0.0049
BW6	99.81 ^a^ ± 0.0033
BW8	100.00 ^a^ ± 0.000
BW10	99.75 ^a^ ± 0.0043
BW14	99.69 ^a^ ± 0.0054
BW1CW1	73.55 ^b^ ± 0.0566
BW2CW2	98.28 ^a^ ± 0.0140
BW4CW4	99.54 ^a^ ± 0.0043
BW6CW6	97.61 ^a^ ± 0.0346
CW8	95.43 ^a^ ± 0.0082
CW10	94.65 ^a^ ± 0.0181
CW14	99.45 ^a^ ± 0.0055

Mean values with the same superscript are not significantly different and vice versa.

**Table 5:** Effect of the type of wax, concentration of wax, and combination of waxes on the slip melting point of RBO oleogels.

Samples	Slip Melting Point (°C)
BW2	25.0 ^g^ ± 0.00
BW4	38.2 ^e^ ± 0.76
BW6	45.0 ^cd^ ± 0.50
BW8	47.0 ^bc^ ± 0.00
BW10	48.2 ^bc^ ± 0.76
BW14	49.8 ^b^ ± 0.76
BW1CW1	25.8 ^g^ ± 0.76
BW2CW2	32.7 ^f^ ± 1.53
BW4CW4	43.5 ^d^ ± 0.50
BW6CW6	46.2 ^cd^ ± 1.26
CW8	30.0 ^f^ ± 2.65
CW10	50.0 ^b^ ± 1.73
CW14	54.0 ^a^ ± 1.00

Mean values with the same superscript are not significantly different and vice versa.

**Table 6:** The influence on the oxidative stability of the RBO oleogels stored under accelerated conditions over a span of 20 days determined using the peroxide value test.

Samples	Peroxide Value (mEq/kg of Sample)				
	Day 4	Day 8	Day 12	Day 16	Day 20
BW2	8.38 ^a^ ± 0.19	20.35 ^ab^ ± 4.21	43.45 ^a^ ± 6.64	57.68 ^a^ ± 4.02	59.79 ^ab^ ± 14.41
BW4	7.26 ^ab^ ± 0.47	22.00 ^a^ ± 1.76	27.57 ^abcd^ ± 8.59	49.65 ^ab^ ± 11.34	72.45 ^a^ ± 11.06
BW6	5.24 ^cd^ ± 0.30	15.29 ^abc^ ± 4.47	22.81 ^bcd^ ± 9.46	40.07 ^bcd^ ± 4.50	45.15 ^bc^ ± 10.23
BW8	8.08 ^a^ ± 0.28	20.58 ^ab^ ± 6.54	29.78 ^abc^ ± 0.58	36.34 ^bcde^ ± 4.85	42.25 ^bcd^ ± 8.67
BW10	5.02 ^de^ ± 0.41	12.91 ^abc^ ± 2.42	20.29 ^bcd^ ± 3.28	25.38 ^def^ ± 3.42	21.40 ^def^ ± 5.82
BW14	2.52 ^f^ ± 0.11	9.07 ^c^ ± 2.04	14.63 ^cd^ ± 3.54	25.43 ^def^ ± 2.73	15.89 ^f^ ± 4.13
BW1CW1	5.57 ^cd^ ± 0.38	18.44 ^abc^ ± 1.72	23.18 ^bcd^ ± 11.00	49.16 ^ab^ ± 6.56	39.56 ^bcde^ ± 9.52
BW2CW2	6.73 ^abc^ ± 0.50	16.14 ^abc^ ± 5.35	20.15 ^bcd^ ± 5.17	43.64 ^abc^ ± 8.00	30.19 ^cdef^ ± 7.04
BW4CW4	6.21 ^bcd^ ± 0.58	10.09 ^bc^ ± 2.97	33.07 ^ab^ ± 3.10	28.69 ^def^ ± 3.77	20.35 ^def^ ± 3.74
BW6CW6	3.51 ^ef^ ± 0.29	10.95 ^bc^ ± 2.96	12.02 ^d^ ± 2.61	22.09 ^ef^ ± 2.84	15.75 ^f^ ± 1.29
CW8	5.83 ^bcd^ ± 0.48	11.00 ^bc^ ± 3.45	11.70 ^d^ ± 5.13	29.25 ^cdef^ ± 1.79	26.90 ^cdef^ ± 9.72
CW10	6.03 ^bcd^ ± 0.40	14.68 ^abc^ ± 1.83	14.44 ^cd^ ± 3.68	27.63 ^def^ ± 1.20	26.42 ^cdef^ ± 2.08
CW14	5.17 ^cde^ ± 1.01	8.89 ^c^ ± 2.60	17.28 ^bcd^ ± 1.40	25.10 ^ef^ ± 1.80	17.21 ^ef^ ± 3.60
Control	5.47 ^cd^ ± 1.22	18.00 ^abc^ ± 2.91	14.15 ^cd^ ± 3.55	15.72 ^f^ ± 2.14	13.75 ^f^ ± 1.26

Mean values with the same superscript are not significantly different and vice versa.

**Table 7:** Effect of the type of wax, concentration of wax and wax combinations on the color of RBO oleogels in comparison to a control sample (margarine).

Samples	Color Parameters		
	L*	a*	b*
BW2	51.16 ^de^ ± 0.11	−0.40 ^bc^ ± 0.03	7.35 ^ef^ ± 0.63
BW4	34.42 ^h^ ± 1.04	−0.71 ^cd^ ± 0.22	6.64 ^f^ ± 0.34
BW6	44.35 ^fg^ ± 0.90	−1.53 ^ef^ ± 0.03	10.54 ^de^ ± 0.41
BW8	52.40 ^d^ ± 0.50	−2.12 ^fgh^ ± 0.15	13.10 ^cd^ ± 0.18
BW10	59.70 ^c^ ± 0.31	−2.75 ^h^ ± 0.06	15.62 ^bc^ ± 0.20
BW14	69.56 ^b^ ± 0.37	−3.52 ^i^ ± 0.07	14.87 ^c^ ± 0.06
BW1CW1	48.11 ^ef^ ± 0.77	0.04 ^b^ ± 0.12	6.44 ^f^ ± 0.36
BW2CW2	45.81 ^fg^ ± 4.25	−0.86 ^cd^ ± 0.41	7.53 ^ef^ ± 2.55
BW4CW4	42.76 ^g^ ± 0.47	−2.68 ^h^ ± 0.26	9.00 ^ef^ ± 0.48
BW6CW6	52.92 ^d^ ± 0.39	−3.81 ^i^ ± 0.14	13.40 ^cd^ ± 0.32
CW8	52.69 ^de^ ± 0.22	−1.07 ^de^ ± 0.21	10.48 ^de^ ± 0.19
CW10	52.32 ^d^ ± 1.27	−1.87 ^fg^ ± 0.50	14.78 ^c^ ± 2.62
CW14	57.19 ^c^ ± 1.75	−2.47 ^gh^ ± 0.16	18.36 ^b^ ± 1.09
Control	86.67 ^a^ ± 0.23	3.94 ^a^ ± 0.04	39.74 ^a^ ± 0.58

Mean values with the same superscript are not significantly different and vice versa.

**Table 8:** Fatty acid compositions of RBO oleogels constructed with BW, CW, and a combination of BW and CW.

Samples	Fatty Acid Composition (%)								
	Myristic Acid	Palmitic Acid	Stearic Acid	Oleic Acid	Vaccenic Acid	Linoleic Acid	Linolenic Acid	Arachidic Acid	Lauric Acid
BW2	0.43 ± 0.11	17.32 ± 1.34	2.05 ± 1.12	39.26 ± 2.78	2.64 ± 0.10	30.67 ± 2.43	2.78 ± 0.11	0.41 ± 0.02	-
BW4	1.33 ± 0.02	18.63 ± 0.87	2.15 ± 0.12	40.84 ± 1.12	1.49 ± 0.12	31.15 ± 1.57	2.50 ± 0.24	0.60 ± 0.16	-
BW6	1.64 ± 0.03	18.59 ± 0.55	2.11 ± 0.34	41.20 ± 1.67	1.27 ± 0.15	31.11 ± 0.78	1.86 ± 0.28	0.58 ± 0.16	-
BW8	0.23 ± 0.00	20.19 ± 1.12	2.28 ± 0.05	42.74 ± 1.78	0.14 ± 0.12	31.71 ± 0.84	2.00 ± 0.13	0.48 ± 0.03	-
BW10	1.75 ± 0.00	18.30 ± 1.01	2.56 ± 0.01	40.51 ± 0.98	1.49 ± 0.33	30.59 ± 1.59	2.28 ± 0.21	0.76 ± 0.21	-
BW14	0.64 ± 0.05	19.68 ± 0.99	2.49 ± 0.07	41.61 ± 0.14	1.05 ± 0.04	31.18 ± 0.44	2.14 ± 0.43	0.55 ± 0.19	-
BW1CW1	0.39 ± 0.01	23.05 ± 0.08	1.93 ± 0.12	39.94 ± 1.56	0.92 ± 0.11	31.20 ± 0.56	1.79 ± 0.54	0.39 ± 0.10	-
BW2CW2	0.15 ± 0.00	20.35 ± 0.18	2.11 ± 0.09	42.50 ± 3.13	0.07 ± 0.10	32.28 ± 1.17	1.88 ± 0.13	0.49 ± 0.05	-
BW4CW4	0.21 ± 0.02	20.46 ± 1.11	2.20 ± 0.01	42.22 ± 1.78	0.11 ± 0.00	32.21 ± 0.67	1.88 ± 0.63	0.50 ± 0.02	-
BW6CW6	0.16 ± 0.02	20.28 ± 1.27	2.28 ± 0.01	42.34 ± 1.55	0.11 ± 0.01	32.23 ± 0.59	1.90 ± 0.14	0.53 ± 0.06	-
CW8	0.85 ± 0.03	18.93 ± 1.17	2.05 ± 0.00	41.29 ± 2.33	1.01 ± 0.01	32.40 ± 2.49	2.15 ± 0.39	0.48 ± 0.13	-
CW10	0.42 ± 0.13	20.41 ± 0.23	2.04 ± 0.00	41.26 ± 0.71	0.95 ± 0.02	32.08 ± 2.13	1.94 ± 0.64	0.47 ± 0.04	-
CW14	1.07 ± 0.01	18.37 ± 0.15	2.01 ± 0.19	41.13 ± 1.11	1.27 ± 0.08	32.41 ± 1.01	2.19 ± 0.77	0.48 ± 0.04	-
RBO	2.43 ± 0.12	17.32 ± 0.17	2.05 ± 0.12	39.26 ± 3.01	2.64 ± 0.36	30.67 ± 0.45	2.78 ± 0.63	0.41 ± 0.02	-
Control	3.31 ± 0.30	35.18 ± 0.67	5.54 ± 1.17	34.20 ± 2.07	2.66 ± 0.37	13.12 ± 1.51	1.05 ± 0.02	0.00 ± 0.02	4.94 ± 0.35

-: Denotes the absence of fatty acids in the sample tested.

**Table 9:** Weight of the respective wax and RBO required for each concentration of oleogel samples (100 g).

Concentration of Oleogel Samples (% *w*/*w*)	Weight of Wax (g)	Weight of RBO (g)
BW2	2.0 BW	98.0
BW4	4.0 BW	96.0
BW6	6.0 BW	94.0
BW8	8.0 BW	92.0
BW10	10.0 BW	90.0
BW14	14.0 BW	86.0
BW1CW1	1.0 BW + 1.0 CW	98.0
BW2CW2	2.0 BW + 1.0 CW	96.0
BW4CW4	4.0 BW + 4.0 CW	92.0
BW6CW6	6.0 BW + 6.0 CW	88.0
CW8	8.0 CW	92.0
CW10	10.0 CW	90.0
CW14	14.0 CW	86.0

BW and CW denote beeswax and carnauba wax, respectively.

## Data Availability

Data will be made available on request.

## Abbreviations

The following abbreviations are used in this manuscript:

BW	Beeswax
CW	Carnauba wax
RBO	Rice bran oil
OBC	Oil binding capacity
DSC	Differential scanning calorimetry
HMWO	High-molecular-weight oleogelator
LMWO	Low-molecular-weight oleogelator
TAG	Triacylglycerol
TPA	Texture profile analysis
PV	Peroxide value
GC	Gas chromatography
FID	Flame ionization detector
